# Hydrothermal Unzipping of Multiwalled Carbon Nanotubes and Cutting of Graphene by Potassium Superoxide

**DOI:** 10.3390/nano12030447

**Published:** 2022-01-28

**Authors:** Apostolos Koutsioukis, Konstantinos Spyrou, Nikolaos Chalmpes, Dimitrios Gournis, Vasilios Georgakilas

**Affiliations:** 1 Department of Materials Science, University of Patras, 26504 Rio, Greece; up1057091@upatras.gr; 2 Department of Materials Science and Engineering, University of Ioannina, 45110 Ioannina, Greece; konstantinos.spyrou1@gmail.com (K.S.); chalmpesnikos@gmail.com (N.C.)

**Keywords:** graphene nanoribbons, graphene dots, nanographene, unzipping, hydrothermal treatment, potassium superoxide

## Abstract

The dual use of potassium superoxide (KO_2_) to unzip multiwalled carbon nanotubes (MWCNTs) and cut graphene under hydrothermal conditions is described in this work. The KO_2_-assisted hydrothermal treatment was proven to be a high-yield method for forming graphene nanoribbons and dots or sub-micro-sized graphene nanosheets. Starting with functionalized MWCNTs, the method produces water-dispersible graphene nanoribbons with characteristic photoluminescence depending on their width. Using pristine graphene, the hydrothermal treatment with KO_2_ produces nanosized graphene sheets and graphene quantum dots with diameters of less than 10 nm. The latter showed a bright white photoluminescence. The effective hydrothermal unzipping of MWNTs and the cutting of large graphene nanosheets is a valuable top-down approach for the preparation of graphene nanoribbons and small nanographenes. Both products with limited dimensions have interesting applications in nanoelectronics and bionanotechnology.

## 1. Introduction

Graphene and carbon nanotubes (CNTs) are the two most well-studied carbon nano allotropes [1]. They are both structured with a hexagonal carbon lattice, which is extended in two dimensions in graphene and wrapped cylindrically in CNTs. Graphene nanosheets and CNTs have been widely studied the last decades in several applications that are related to their properties, such as electrical and thermal conductivity, optical transparency, chemical reactivity, and mechanical strength [2,3,4,5]. Thanks to their nanosized thickness, they have been often used as substrates in bioapplications and nanoelectronics [6,7,8,9,10,11,12]. One of the most challenging demands with regard to their application is the control of their size and dimensions. Therefore, their electrical and optical properties depend, among other things, on the size and the thickness of the graphene or CNTs. Bioapplications, such as drug or gene delivery, usually also need much smaller graphene nanosheets than those usually isolated from graphite [13,14]. On the other hand, a new graphenic nanostructure has been developed over the last few years called graphene nanoribbons (GNRs), and it is considered to be an attractive material, since it combines planar graphenic structures, including graphene nanosheets, and CNTs with narrow widths and very high aspect ratios [15]. Their electrical properties are strongly related to their width. Narrow GNRs appear to have a nonzero bandgap and can be used in field-effect transistors, while broad GNRs are conductive like graphene. Several studies have appeared in the literature describing their applications in quantum electronics, and in bionanotechnology with GNRs [15,16,17,18].

GNRs can be made, among other ways, by the unzipping of CNTs, which means making a cut in the longitudinal direction. Usually, their width ranges between 10 and 100 nm, and their lengths are related to that of the starting CNTs [19,20,21]. The oxidative treatments of CNTs with a sulfuric acid solution of potassium permanganate [19,20,22], potassium vapors [19], and palladium chloride ions (PdCl_4_^−2^) [23] are characteristic procedures for the top-down approaches of nanoribbon formation. Several researchers have proposed, as a mechanism, the formation of manganate ester in lines, which is followed by the dissociation of carbon–carbon bonds through an oxidative or a reductive reaction [19]. Bottom-up methods have been also developed, combining aromatic molecules and specific organic reactions. In this way, narrow nanoribbons are constructed with widths of less than 10 nm [24,25,26,27].

Similar cutting methods have been also developed recently for graphene, leading to small-sized graphenic pieces with interesting optical properties: the so-called graphene quantum dots (GQDs) [28,29,30]. Their size ranges between 2 and 10 nm and they appear to exhibit a characteristic fluorescence emission upon excitation. Although the cutting of graphene or CNTs leads to different nanostructures, the proposed mechanism of cutting the common hexagonal carbon lattice is similar in both cases [31,32]. Hydrothermal treatment is a relatively new, very promising procedure that has been often used with graphene and CNTs and their derivatives. The limitation of water dispersibility is a disadvantage for the use of pristine graphene or carbon nanotubes; however, graphene oxide (GO), which is easily dispersible in water, is very often used [29,31,32,33]. Hydrothermal heating with potassium superoxide (KO_2_) has been proven to be an effective method to produce GQDs from GO [34].

In this article, we explore the ability of KO_2_ to unzip functionalized water-dispersible multiwalled carbon nanotubes (MWCNTs) under hydrothermal conditions to create GNRs. Furthermore, we show that the same reaction is highly effective, as well, at cutting pristine graphene nanosheets to produce nanographenes and GQDs. The method is simple and eco-friendly, and can be performed at a large scale with good yield.

## 2. Materials and Methods

Potassium superoxide purchased from Sigma-Aldrich, MWNT-f-OH, were prepared according to [35]. Graphene was purchased from XG Science.

### 2.1. Instrumentation

FTIR spectra were obtained with an ATR technique on a Fourier transform spectrometer (IRTracer-100, Shimadzu Europa GmbH, Duisburg, Germany). Raman spectra were collected both with a Raman System T-64000 and a LabRam-HR Mirco Raman spectrometer (Horiba, NJ, USA) using a laser excitation line at 514nm. The laser power was 1.082 mV. XRD was conducted with a D8 Advance Bruker diffractometer (Bruker AXS, Karlsruhe, Germany) using a CuKa (lD 1.5418) radiation source (40 kV, 40 mA) and a secondary beam graphite monochromator. Diffraction patterns were recorded in the 2-theta (2′) scale from 2° to 80°, in steps of 0.02° and with a counting time of 2 s per step. The optical absorption spectra were obtained with a Hitachi Digilab, Model U2800-Double Beam-UV/Vis (Tokyo, Japan).

Surface roughness was measured by using AFM atomic force microscopy (AFM) images that were collected in tapping mode with a Bruker Multimode 3D Nanoscope (Ted Pella Inc., Redding, CA, USA) using a microfabricated silicon cantilever type TAP-300G, with a tip radius of <10 nm and a force constant of approximately 20–75 N m^−1^. The Si wafers (P/Bor, single-side polished, Si-Mat) used in the AFM imaging were cleaned before use for 20 min in an ultrasonic bath (160 W) with water, acetone (≥99.5% Sigma-Aldrich, St. Louis, MO, USA), and ethanol (≥99.5% Sigma-Aldrich, St. Louis, MO, USA).

X-ray photoelectron spectroscopy (XPS) measurements were performed in an ultra-high vacuum at a base pressure of 6 × 10^−9^ mbar with a SPECS GmbH spectrometer equipped with a monochromatic Mg Kα source (hv = 1253.6 eV) and a Phoibos-100 hemispherical analyzer (Berlin, Germany). Spectral analysis included a Shirley background subtraction and peak separation using Gaussian–Lorentzian functions in a least squares fitting software package (Winspec) developed at the LISE laboratory, University of Namur, Belgium.

The sheet resistance of the samples was measured on circular spots made from the samples after being compressed at 2 tn by a 4-point probe system (Pro4 Resistivity System, Lucas Labs, Gilroy, CA, USA) and Keithley 2400 Source Meter. The thickness was estimated to be 5 μm [35]. 

### 2.2. Sample Preparation

For this step, 5 mg of catechol-functionalized MWCNTs [35] or pure graphene (XG Science) were dispersed in 6 mL H_2_O by sonication for 30′, and then 50 mg of KO_2_ was added, followed by stirring for another 30′. After that, the mixture was heated hydrothermally overnight at 185 °C in an autoclave Teflon. 

Carbon nanotubes: The product of the hydrothermal reaction was separated in two parts by precipitation: the supernatant (NR_1_) and the precipitate (NR_2_). The brown-to-yellow-colored supernatant was purified with dialysis membrane, while the precipitate was washed and isolated by centrifugation (15,000 rpm/15′) and redispersed in water. The supernatant was left for a week on a filtration membrane to remove by-products. 

Graphene nanosheets: The unreacted material or large graphene nanosheets were separated by precipitation. The liquid phase was left overnight and the precipitate that formed was separated by centrifugation (15,000 rpm/15′) (NG). The final brown-to-yellow supernatant was purified with a dialysis membrane (GQDs).

## 3. Results

### 3.1. Unzipping of MWNTs

**Figure 1:**
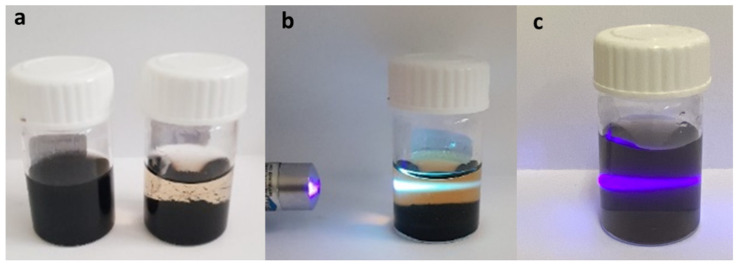
(**a**) MWCNT-f-OH in water before (left) and after (right) the hydrothermal treatment. (**b**,**c**) Excitation with violet laser lamp (405 nm) of the supernatant sample NR_1_ showing blue emission (**b**), and the precipitate sample NR_2_ redispersed in water (**c**) showing no emission. The violet light is due to the Tyndall effect of the dispersed NR_2_.

Functionalized MWCNTs (MWCNT-f-OH) dispersed in water were mixed with an excess of KO_2_ in water and heated hydrothermally overnight at 185 °C. The mixture of the hydrothermal heating after sedimentation was separated in two parts: the supernatant (NR_1_) and the precipitate (NR_2_) (see Figure 1a). The brown-to-yellow-colored supernatant was purified using a dialysis membrane, while the precipitate was washed several times with water and isolated by centrifugation. The first evidence that indicated that different products were dispersed in the separated parts, apart from the color, was the photoluminescence of the NR_1_ sample, as shown in Figure 1b, upon excitation with a violet laser lamp (405 nm). The sample NR_2_ (diluted in water) showed no fluorescence under the same excitation (Figure 1c).

**Figure 2:**
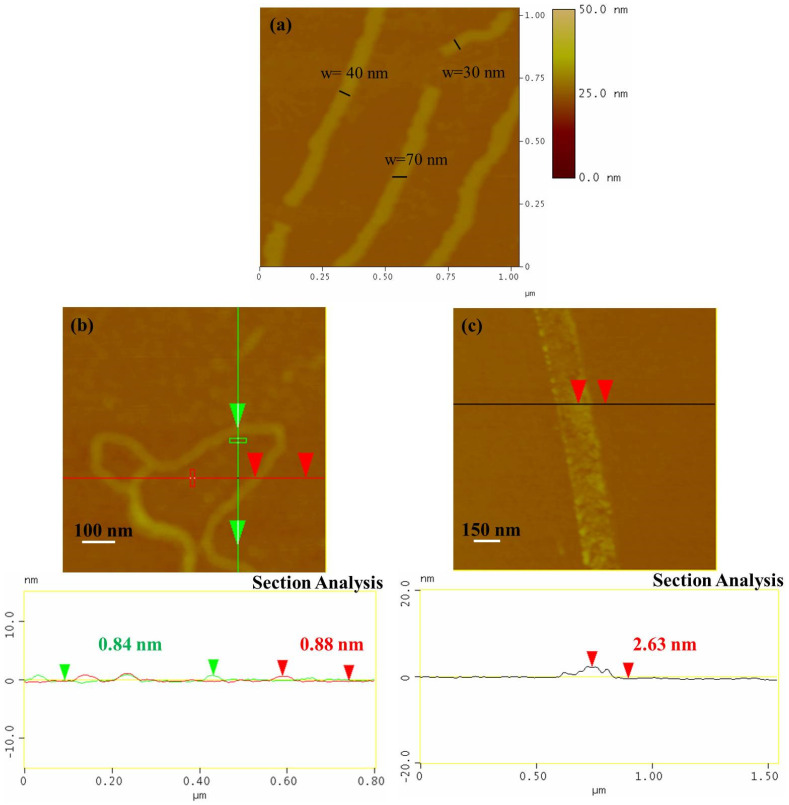
AFM images from the sample NR_1_ (**a**) and the sample NR_2_ (**b**,**c**).

**Figure 3:**
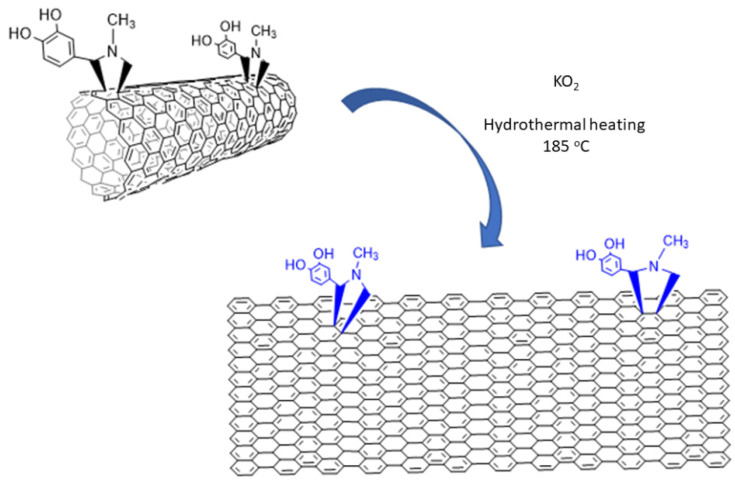
Unzipping of MWCNT-f-OH by KO_2_.

*AFM images*. The AFM images of the sample NR_1_ reveal that it contains elongated single-layered nanoribbons with a thickness of 0.86 nm or some thicker few layers nanoribbons (~4 nm) and a mean width close to 40 (see Figure 2a,b and Figure S1). The lower thickness indicates the formation of the thinnest one-layer nanoribbons. The precipitate sample, NR_2_, was also dispersible in water, creating a gray-to-black-colored dispersion, and it also contains elongated nanoribbons, with a larger width between 100 and 150 nm and a thickness between 2 and 3 nm, as revealed by the AFM images (see Figure 2c and Figure S2). Considering that a nanoribbon is formed by the unzipping of a MWCNT, it can be reasonably supposed that, here, the starting MWCNTs had an external layer with diameter between 30 and 50 nm that was unzipped, leading to the larger GNRs of sample NR_2_, while the internal layers that formed the narrower GNRs of sample NR_1_ behaved similarly (see Figure 3). The fact that the NR_2_ samples were less dispersible than the starting MWCNT-f-OH and precipitate in the reaction mixture (see Figure 1a) could be attributed to their different structure. MWCNT-f-OH is cylindrical, and the external surface is enriched by hydrophilic catechol groups that remarkably improve their dispersibility in water (see Figure 3). On the other hand, NR_2_ are plain structures with two hydrophobic aromatic surfaces, where only a part of them is decorated with catechols. The lower hydrophilic character of NR_2_ is due to the oxygen groups that formed at the edges after the hydrothermal scissoring.

**Figure 4:**
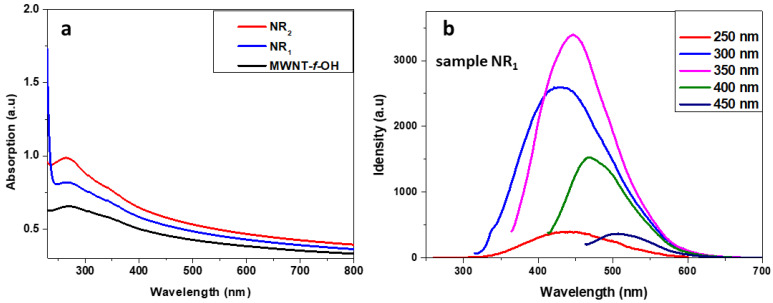
(**a**) UV–Vis spectra of samples NR_1_, NR_2_, and MWCNT-f-OH; (**b**) PL spectra of sample NR_1_ at several excitation wavelengths.

*Photoluminescence*. The photoluminescence (PL) of the samples NR_1_ and NR_2_ was evaluated using PL spectroscopy, as shown in Figure 4. The UV–Vis spectra of samples NR_1_ and NR_2_ were similar and show a broad absorption with a maximum at 263 nm, which corresponds to the π,π* transition and a very broad, and weak absorption around 350 nm that corresponds to the n,π* transition (see Figure 4a). A similar absorption curve was recorded by the MWCNT-f-OH sample. Although both NR_1_ and NR_2_ samples have similar UV–Vis absorption curves, only the sample NR_1_ showed photoluminescence. Under excitation with 250 to 450 nm, sample NR_1_ showed an excitation that depended on luminescence, with the maximum ranged between 430 and 500 nm. The most intense luminescence band has a λ_max_ at 446 nm under excitation with 350 nm. The sample NR_2_ showed no photoluminescence, nor did the starting ΜWCNT-f-OHs.

Considering the AFM images, the main difference between the two samples was the width of the nanoribbons. Hence, it is reasonably supposed that photoluminescence was determined by the width of the carbon nanoribbons [25]. In the literature, nanoribbons that have been detected with photoemission were mostly produced by bottom-up methods and their width was limited to 10 nm [24,25,26,27].

**Figure 5:**
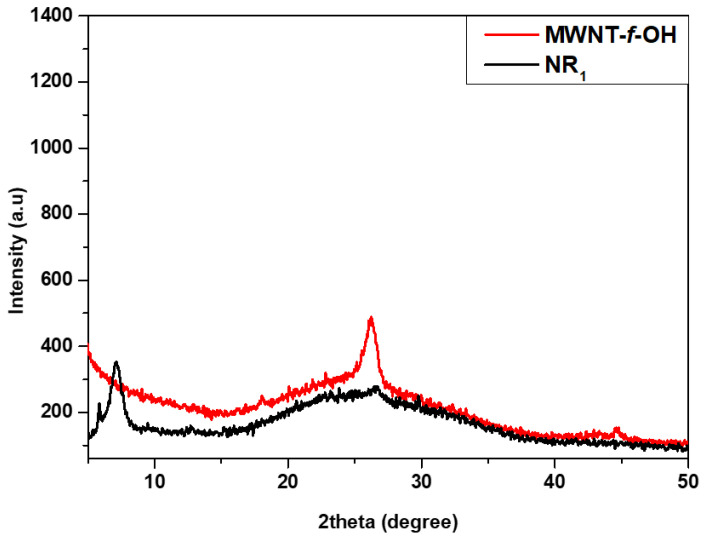
XRD diffraction pattern of MWCNT-f-OH and carbon nanoribbons NR_2_.

*XRD and Raman*. The XRD pattern of the MWCNT-f-OHs showed a characteristic peak at 26° that corresponds to the 0.34 nm interlayer distance, which is characteristic between the graphenic walls of MWCNTs (see Figure 5). A similar peak did not appear in the XRD pattern of the product NR_2_, indicating the effective unzipping of MWCNTs. A new peak that appeared at 7° corresponds to an interlayer distance of 1.26 nm, and could be attributed to the catechol pyrrolidine groups and the introduction of oxygen-containing groups on the edge of each layer, similar to GO [36]. 

**Figure 6:**
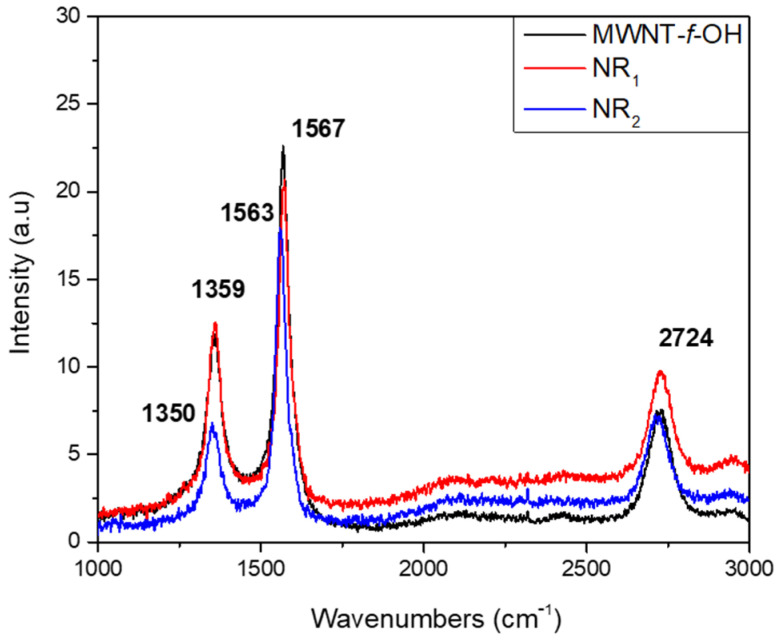
Raman spectra of MWCNT-f-OH and products NR_1_ and NR_2_ of carbon nanoribbons.

The Raman spectra of products NR_1_ and NR_2_ were like that of MWCNT-f-OH (see Figure 6). The characteristic D and G bands of MWCNT-f-OH and sample NR_1_ are located at 1359 and 1567 cm^−1^, respectively, where the same bands for the sample NR_2_ are blue shifted to 1350 and 1563 cm^−1^, respectively. The 2D band was located at 2724 cm^−1^ in the spectra of both the products and the MWCNT-f-OH. The relatively high value of the I_D_/I_G_ ratio of MWCNT-f-OH (0.77) is attributed to the added functional groups. Comparing the values for the I_D_/I_G_ ratio, which is 0.36 and 0.64 for the spectra of products NR_2_ and NR_1_, respectively, the much lower value recorded for NR_2_ reflects the increased ratio between the sp^2^ carbon atoms at the core and the sp^3^ carbon atoms at the edges, due to the much larger width of the NR_2_ product [22].

**Figure 7:**
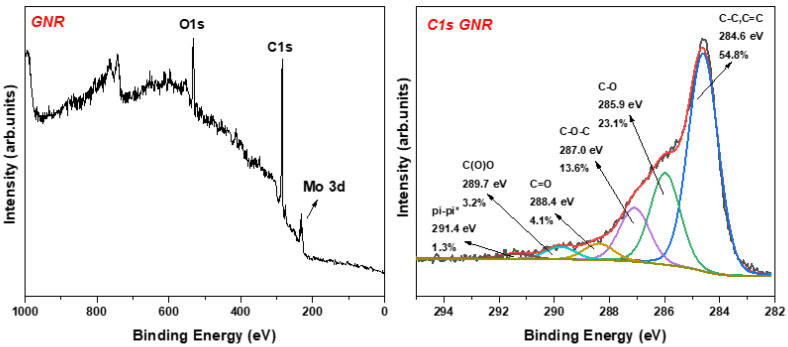
(**Left**) XPS survey of NR_2_, where the Mo peak is attributed to the substrate at which the sample was drop casted; (**Right**) C1s photoelectron peak of NR_2_.

The XPS analysis of the sample NR_2_ showed the different functional chemical groups (see Figure 7). The main fitted peak at 284.6 eV is due to the basic carbon frame created by sp^2^ and sp^3^ hybridization. A second intense peak at 285.9 is attributed to the C-OH groups, representing 23.1% of the whole carbon area. The remaining peaks are associated with the epoxy (13.6%), carbonyl (4.1%), and carboxyl (3.2%) groups. The pi–pi* interactions may derive from the few layers nanoribbons while interacting together via aromatic systems.

The electrical conductivity of large nanoribbons NR_2_ was estimated to be 1075 S m^−1^ (sheet resistance: 186 Ohm sq^−1^) and increased to 1835 S m^−1^ (sheet resistance: 109 Ohm sq^−1^) after the reduction of the product with NaBH_4_. The conductivity of the nanoribbons was lower than that of the starting MWCNT-f-OH, which was measured to be 2940 S m^−1^ (sheet resistance: 68 Ohm sq^−1^); however, it was remarkably higher compared with similar nanoribbons in the literature and suitable for applications in nanoelectronics. In the literature, several procedures for the unzipping of MWCNTs have been described. Unfortunately, few reports presented a common description of the nanoribbon product, which usually contains the dimensions, dispersibility, conductivity or sheet resistance, yield, etc. In Table 1, the most important characteristics of some common methods for the preparation of carbon nanoribbons—mainly by unzipping MWNTs—are presented. The KO_2_-assisted hydrothermal treatment is one of the most eco-friendly, low-cost, and high-yield methods. Apparently, it is the only one that can produce simultaneously narrow nanoribbons with intense photoluminescence and wide nanoribbons with an electrical conductivity between the highest points, especially after reduction.

### 3.2. Cutting of Graphene Nanosheets

**Figure 8:**
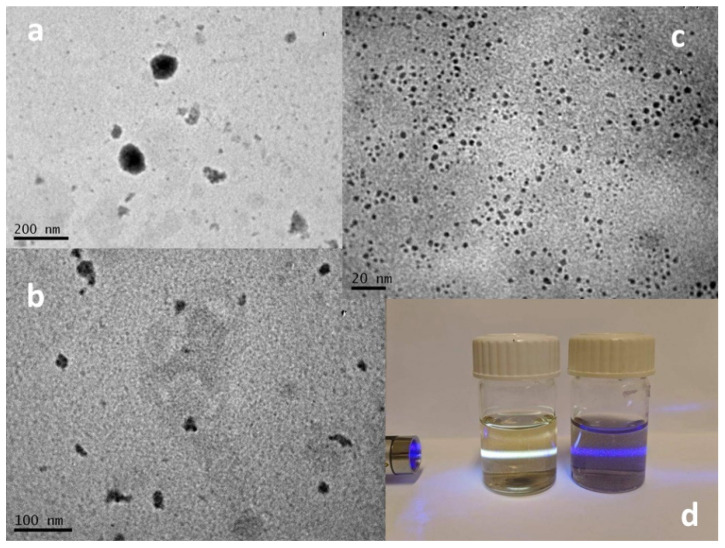
TEM images of (**a**,**b**) nanographenes (NGs) and (**c**) GQDs. (**d**) Photo of GQDs and NGs dispersed in water. GQDs emit white light after excitation with violet laser light, while NGs do not emit light.

The same reaction was then applied to pristine graphene nanosheets. Graphene nanosheets were dispersed in water, and their hydrothermal treatment with KO_2_ was also performed successfully. The products of the reaction, after the removal of the unreacted material or the large graphene nanosheets, were also divided by the material that was dispersed in the supernatant and the precipitate. As revealed by TEM analysis (see Figure 8), the precipitate mainly consisted of large nanographenes (NG) with diameters between 50 and 100 nm, and the supernatant created by graphene quantum dots (GQD) had a diameter that ranged between 2 and 6 nm. GQDs were easily dispersible in water and had a characteristic photoluminescence, in contrast with the larger NGs, which are less dispersible in water and without photoluminescence.

**Figure 9:**
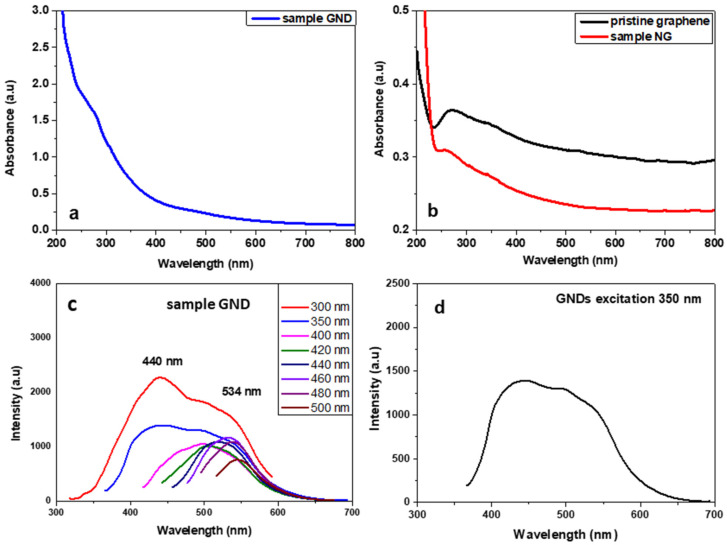
UV–Vis spectra of product GND (**a**), product NGs and pristine graphene (**b**), and PL spectra of GQD sample after excitation at several wavelengths (**c**). Broadband emission after excitation of GNDs with 350 nm (**d**).

The UV–Vis absorption spectrum of GQDs showed a wide band that starts from 800 nm and is continuously increased until the end of the spectrum measurement at 250 nm. Two broad shoulders can be also observed at 280–300 nm (the π,π* transition) and between 450 and 550 nm (the n,π* transition). The photoluminescence spectra of the GQD sample at several excitation wavelengths showed two different luminescence bands. The first with λ_max_ around 440 nm was recorded after excitation under 300 and 350 nm, and the second was an excitation that depended on luminescence, with a maximum emission between 500 and 550 nm after excitation under 400 to 500 nm (see Figure 9). Interestingly, after the excitation with 300–350 nm, GNDs emitted a broad-band white light that extended from 400 to 600 nm because of the co-existence of the two different bands (see Figure 9b). The different emission bands here could be related to the size of the GNDs. According to Ajayan et al., the energy gap of the GNDs decreases with the increasing size [39]. Thus, the broad emission band is a result of the co-existence of GNDs with sizes between 2 and 6 nm, as revealed also by the TEM image (see Figure 8c).

**Figure 10:**
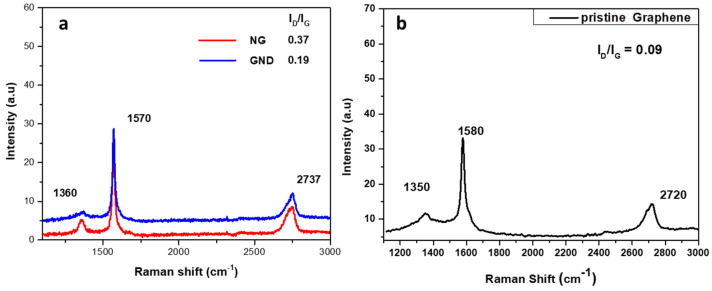
Raman spectra of (**a**) NG and GQDs; (**b**) pristine graphene.

The Raman spectra of the two products showed the characteristic peaks of the D, G, and 2D bands at 1360, 1570, and 2737 cm^−1^, respectively. Both products have an increased I_D_/I_G_ ratio compared to that of pristine graphene nanosheets, due to the increased number of sp^3^ carbon atoms at the edges of the much smaller GQDs and NGs. The difference between the I_D_/I_G_ ratio of the two products, as shown in Figure 10, can be reasonably attributed to the fact that NGs have more sp^3^ carbon atoms due to the existence of defects at the core apart from the edges.

**Figure 11:**
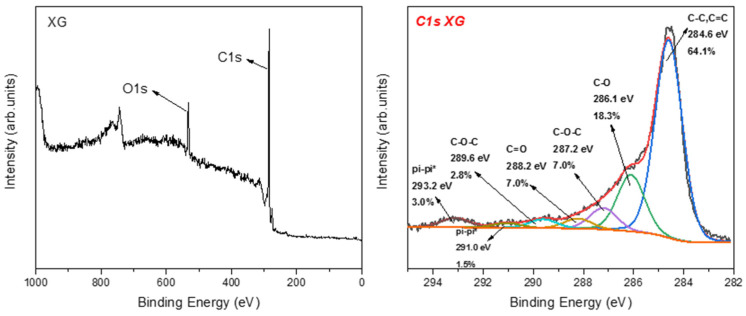
(**Left**) XPS survey of NG and (**right**) C1s photoelectron peak of Ng.

The XPS analysis of nanographene showed different types of oxygen functional groups (see Figure 11). The main peak at 284.6 eV is due to C–C and C=C bonds, while the peak at 286.1 is ascribed to the C–OH bonds, representing 18.3% of the carbon area. The less intense peaks at 287.2 eV (7.0%), 288.2 eV (7.0%), and 289.6 eV (2.8%) represent the C–O–C, C=O, and COOH groups, respectively. Finally, the contribution of pi–pi* interactions was observed; this is characteristic for carbon in aromatic compounds between π electron systems, such as aromatic rings.

The effectiveness of the KO_2_-assisted hydrothermal treatment for both substrates was indicated by the relatively high yield from unzipping MWCNT-f-OH or cutting graphene nanosheets. For the first procedure, the yield was above 90%, as indicated by the limited number of unreacted MWCNT-f-OH that was observed in the TEM images. As regards the hydrothermal cutting of graphene, the KO_2_ assistance resulted in a remarkable yield that reached 50%. The KO_2_-assisted hydrothermal treatment has proven to be an effective top-down method for the preparation of nanosized carbon structures from different graphenic precursors, such as carbon nanotubes, pristine graphene, or graphene oxide [34]. Preliminary results showed that carbon black could be also a precursor. Although the emissive NR1 and GQDs are very interesting nanomaterials, which potentially could be used in optoelectronic applications, the main products here are the wide, highly conductive nanoribbons NR_2_ and nanographenes NG (50–100 nm). The first could be used for the preparation of transparent conductive films in several applications, such as photovoltaics, smart screens, etc. Finally, nanographenes seems to have the suitable size to be used in bioapplications, such as drug delivery, biosensing, etc.

**Figure 12:**
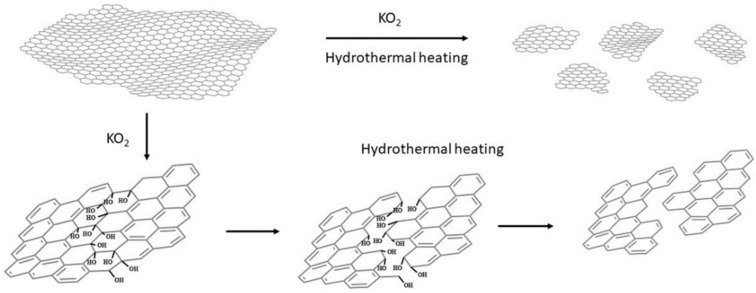
The proposed mechanism of the cutting of graphene nanosheets.

*The mechanism*. The mechanism of this effective cutting of graphenic layers or unzipping carbon nanotubes has been already proposed by Zhao et al., who showed that KO_2_ hydrothermally cut GO into very small pieces, thus producing GQDs with yellow PL emission [34]. It is known that KO_2_ in water produces an intermediate superoxide anion O_2_^−^, and finally OH^−^ and O_2_. The mechanism proposed by Zhao et al. is based on the existence of epoxy groups along lines on the GO surface or the formation of new lines of epoxy groups by the strongly oxidative superoxide anion O_2_^−^, and the dissociation of the underlying C-C bonds by OH^−^ during the hydrothermal heating. In our work, functionalized MWCNTs and pristine graphene—without epoxy groups—were used, which means that epoxy lines should be formed exclusively during the KO_2_ treatment of the starting material (see Figure 12).

**Figure 13:**
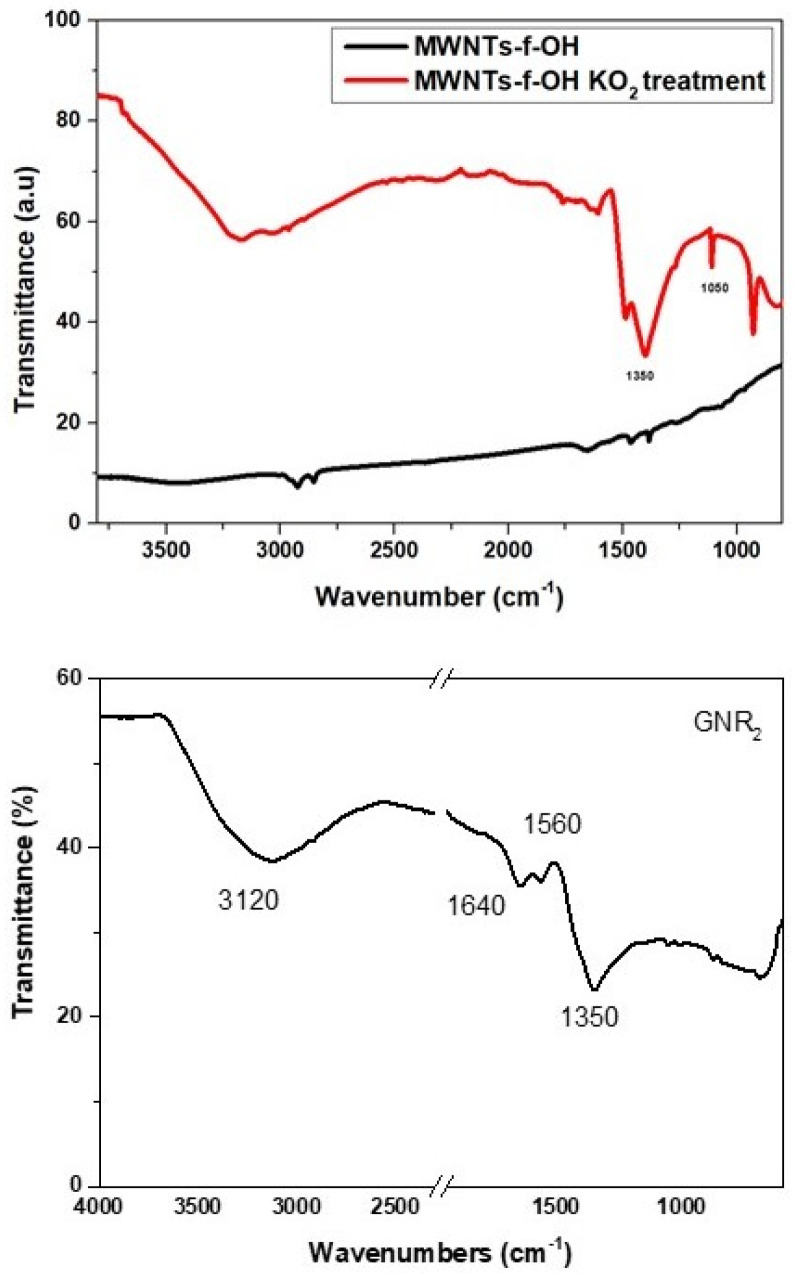
IR spectra of the graphene or MWCNT-f-OH after the treatment with KO_2_ in water.

To support this hypothesis, pristine graphene and MWCNT-f-OH were mixed with an excess of KO_2_ in water under sonication for a few minutes, and the product was separated and washed repeatedly to remove by-products. The KO_2_-treated graphene and MWCNT-f-OH were characterized by FTIR spectroscopy, which showed in both cases the formation of a strong band at 1350 cm^−1^ and a broad band at 3150–3200 cm^−1^ (see Figure 13).

The latter can be attributed to OH stretching vibrations and the former to C-O bending vibrations or epoxy groups. The lack of any other characteristic peak in the FT-IR spectra of both samples indicates the formation of epoxy groups, which probably was partly hydrolyzed to C-OH during the KO_2_ treatment of graphene or MWCNT-f-OH, and before the hydrothermal heating in the presence of OH^−^, which favors the cutting or unzipping of the epoxy or hydroxy functionalized graphene or MWCNT-f-OH surface.

## 4. Conclusions

The hydrothermal treatment of hydrophilic-functionalized MWCNTs with KO_2_ leads to the effective unzipping and production of GNR. The same reaction was successfully applied to pristine graphene, creating nanographenes and GQDs. In both cases, there is a hydrophilic emissive nanostructure that was isolated by the water phase—narrow GNRs or GQDs—and a less hydrophilic, non-emissive part that isolated a precipitate that contains larger GNRs or nanographenes. Interestingly, narrow GNRs and GQDs are emissive nanostructures and, significantly, GQDs emit white light after excitation at 350 nm.

## Figures and Tables

**Table 1:** Comparison of various method for the formation of nanoribbon.

	Method	Width of Nanoribbons	Conductivity	Yield	Ref
MWNT	H_2_SO_4_–KMnO_4_	>100 nm	poor	nearly 100%	[19]
MWNT	Air at 500 °C/sonication	10–30 nm	good	2%	[21]
Carbon black	HNO_3_/90 °C	30 nm		66%	[37] *
MWNT	KOH/900 °C		628 S m^−1^	53%	[38]
MWNT-f-OH	KO_2_ 120 °C hydrothermal	30–150 nm	1730 S m^−1^	>90%	**

* Nanoribbons with photoluminescence. ** This work.

## Data Availability

Not applicable.

## Supplementary Materials

The following are available online at https://www.mdpi.com/article/10.3390/nano12030447/s1, Figure S1: AFM images of GNR from sample NR_1_; Figure S2. AFM images of GNR from the sample NR_2_.
