# Supplementary material for: Hydrothermal Unzipping of Multiwalled Carbon Nanotubes and Cutting of Graphene by Potassium Superoxide
Source: Nanomaterials (Basel). 2022 Jan 28;12(3):447. doi: 10.3390/nano12030447 (PMC8839989; doi:10.3390/nano12030447)
Supplement: Supplementary file 1 [file nanomaterials-12-00447-s001.zip › nanomaterials-1546012-supplementary.pdf]

Supplementary Materials

Hydrothermal Unzipping of Multiwalled Carbon Nanotubes and Cutting of Graphene by Potassium Superoxide

Apostolos Koutsioukis [1], Konstantinos Spyrou [2], Nikolaos Chalmpes [2], Dimitrios Gournis [2,*] and Vasilios Georgakilas [1,*]

[1] Department of Materials Science, University of Patras, 26504 Rio, Greece; up1057091@upatras.gr
[2] Department of Materials Science and Engineering, University of Ioannina, 45110 Ioannina, Greece; konstantinos.spyrou1@gmail.com (K.S.); chalmpesnikos@gmail.com (N.C.)
* Correspondence: dgourni@uoi.gr (D.G.); viegeorgaki@upatras.gr (V.G.)

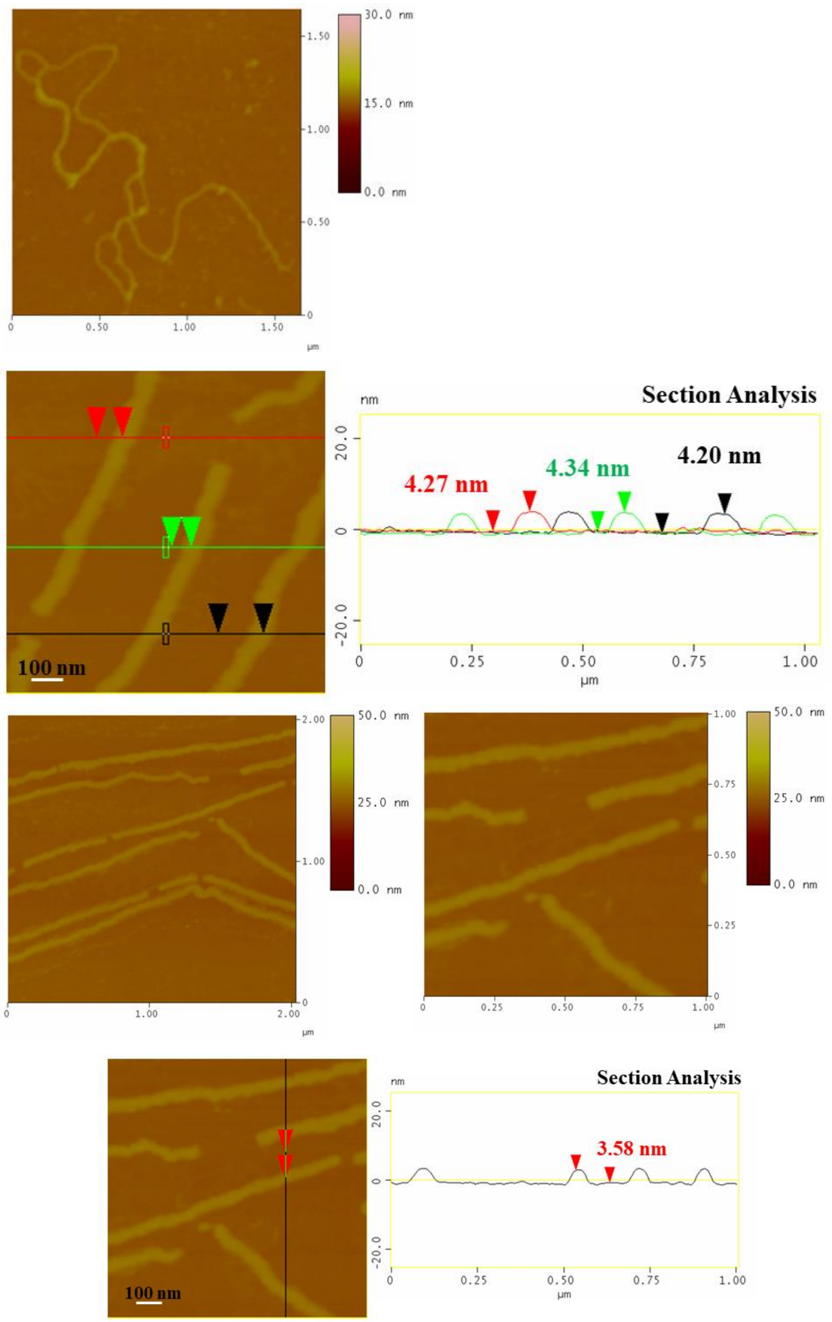
30.0 nm
15.0 nm
0.0 nm
1.50
1.00
0.50
0
0.50
1.00
1.50
µm
Section Analysis
nm
20.0
0
-20.0
4.27 nm
4.34 nm
4.20 nm
100 nm
0
0.25
0.50
0.75
1.00
µm
50.0 nm
25.0 nm
0.0 nm
2.00
1.00
0
µm
50.0 nm
25.0 nm
0.0 nm
1.00
0.75
0.50
0.25
0
µm
Section Analysis
nm
20.0
0
-20.0
3.58 nm
100 nm
0
0.25
0.50
0.75
1.00
µm

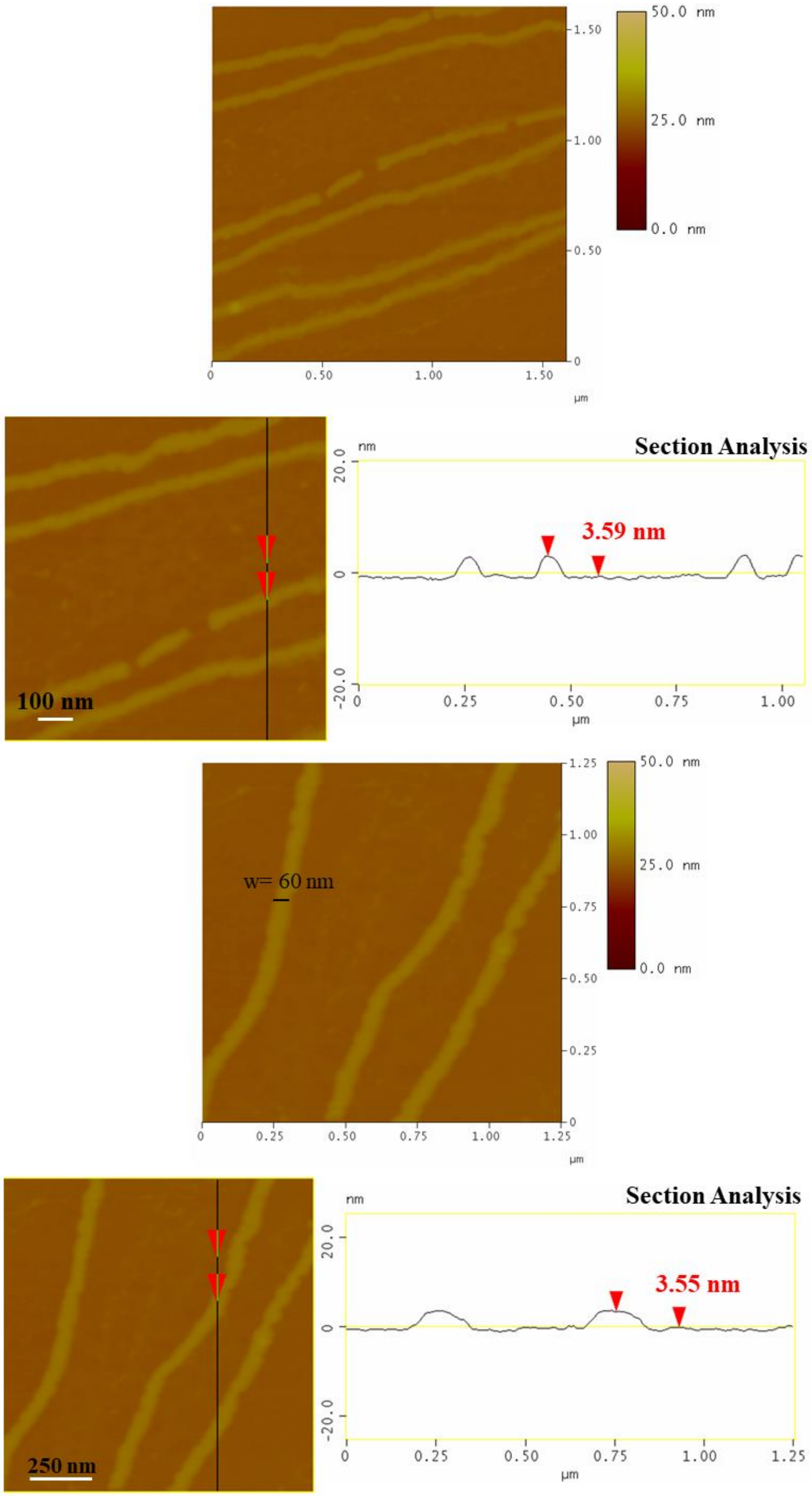

Figure S1. AFM images of GNR from sample NR_1.

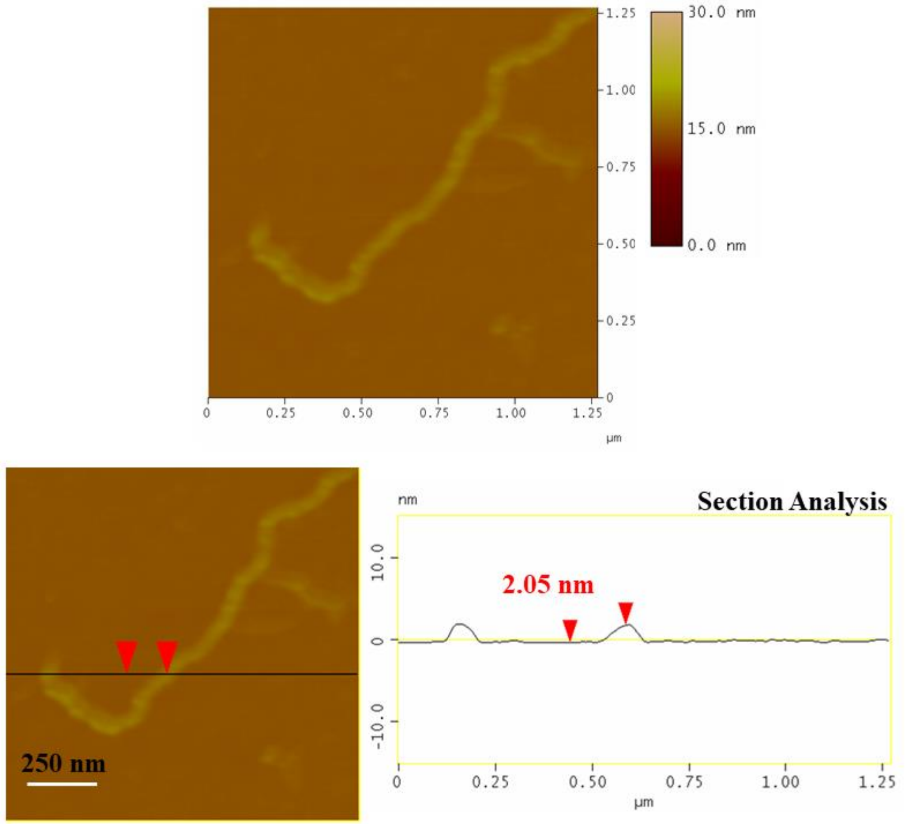

Figure S2. AFM images of GNR from the sample NR_2.
